# The Action of Urine on the Tissues

**Published:** 1870-09

**Authors:** 


### The Action of Urine on the Tissues.

Professor G. Simon, *Deutshe Klinik,* has made experiments on this subject. He remarks that it has been a dogma in surgery, that urine, whatever may be its reaction, has a destructive action on tissues not protected by an epithelial covering. He injected subcutaneously in rabbits pure acid urine. It was absorbed without any apparent bad effect. Operation wounds, moistened with fresh urine, healed by primary intention. When ammoniacal urine was injected, even though it had been filtered, abscesses were formed, and the skin over them became gangrenous. In view of these results, the gangrene which appears so rapidly in cases of infiltration of urine, must be ascribed to the mechanical action of the fluid driven forcibly among the tissues, so as to tear or compress the blood-vessels. In plastic operations on the urinary or sexual organs, therefore, it is unnecessary to leave a catheter in the bladder, so long as the urine is acid, whilst such operations should not be performed, if possible, when the reaction is alkaline.—*Medical Press. Dominion Medical Journal.*

